# O9.5. ABERRANT DOPAMINE SYSTEM FUNCTION REVERSED BY THE OREXIN RECEPTOR ANTAGONIST TCS1102 IN A RODENT MODEL OF SCHIZOPHRENIA

**DOI:** 10.1093/schbul/sby015.249

**Published:** 2018-04-01

**Authors:** Stephanie Perez, Daniel Lodge

**Affiliations:** University of Texas Health Science Center at San Antonio

## Abstract

**Background:**

Aberrant regulation of dopamine system function is thought to contribute to psychosis in schizophrenia patients; however, the brain regions associated with this dysregulation have not been conclusively demonstrated. We have recently demonstrated that medium spiny neurons in the nucleus accumbens (NAc) receive convergent input from the ventral hippocampus (vHipp) and paraventricular nucleus of the thalamus (PVT). Furthermore, inactivation of either the vHipp or PVT is sufficient to reverse aberrant dopamine system function in rodent models of schizophrenia. Using, chemogenetic experiments we now provide conclusive evidence that the thalamic input to the NAc plays a role in the regulation of dopamine neuron activity. These data demonstrate that the vHipp and thalamus (specifically the PVT) work in concert to regulate VTA dopamine neuron population activity. Such data are important as they provide evidence that thalamic abnormalities may contribute to the aberrant dopamine system function observed in schizophrenia and suggest that the PVT may be a novel site for intervention in psychosis. To examine this, we explored the orexin system, which is known to provide a dense innervation of the PVT.

**Methods:**

Pregnant Sprague Dawley (SD) rats were treated on gestational day (GD) 17 with either methylazoxymethanol acetate (MAM; 22 mg/kg, i.p.) or saline. For Poly I:C, pregnant dams were treated on GD12 (7.5 mg/kg Poly I:C or saline). Male pups weaned on post-natal day 21 in groups of 2–3 until adulthood (>60 days). For chemogenetic experiments, normal SD rats were bilaterally micro-injected with AAV2 vectors (Addgene) expressing hm3D(Gq)(pAAV-h8yn-HA-hm3D(Gq)-mcherry; 0.5μL) into the PVT or mPFC. Control rats were administered the viral vector lacking the hm3D encoding gene. Prior to testing, CNO (0.75ul; 300uM) was injected into the nucleus accumbens. In vivo extracellular recordings were performed to measure dopamine neuron activity in the VTA. Spontaneously active VTA dopamine neurons were recorded using previously established electrophysiological criteria.

**Results:**

NMDA activation of the PVT induces a significant increase in VTA dopamine neuron population activity. MAM- and Poly I:C-treated rats (both verified rodent models of schizophrenia) consistently display aberrant VTA dopamine neuron population activity, which is restored by pharmacological inactivation of the PVT with TTX. Chemogenetic activation of PVT neurons projecting to the mPFC do not affect VTA dopamine neuron activity; however, activation of PVT neurons projecting to the nucleus accumbens induces a significant increase in dopamine neuron population activity. This effect can be replicated in rats that receive microinjections of the endogenous orexin peptide A or B into the PVT. Consequently, dopamine neuron function can be restored in MAM-treated rats that received a systemic injection of the orexin peptide antagonist TCS 1102.

**Discussion:**

We now demonstrate that orexin receptors are expressed on PVT neurons projecting to the NAc and may serve as a substrate for pharmacological manipulation of this pathway. Here, we provide evidence that both systemic and intracranial (PVT) administration of the orexin receptor antagonist, TCS1102, can normalize aberrant dopamine system function in a rodent model of schizophrenia. Collectively, these data suggest that targeting orexin signaling in the thalamus, specifically, the PVT, may represent a novel site of intervention for psychosis associated with schizophrenia.

